# Draft Genome Sequence of *Pseudomonas putida* CBF10-2, a Soil Isolate with Bioremediation Potential in Agricultural and Industrial Environmental Settings

**DOI:** 10.1128/genomeA.00670-16

**Published:** 2016-07-14

**Authors:** Rupa Iyer, Ashish Damania

**Affiliations:** a Center for Life Sciences Technology, College of Technology, University of Houston, Houston, Texas, USA; b Department of Pediatrics-Tropical Medicine, Baylor College of Medicine, Houston, Texas, USA

## Abstract

*Pseudomonas putida* CBF10-2 is a microorganism isolated from farmland soil in Fairchild, TX, found to degrade high-impact xenobiotics, including organophosphate insecticides, petroleum hydrocarbons, and both monocyclic and polycyclic aromatics. The versatility of CBF10-2 makes it useful for multipurpose bioremediation of contaminated sites in agricultural and industrial environments.

## GENOME ANNOUNCEMENT

*Pseudomonas putida* is a Gram-negative gammaproteobacterium ubiquitous to contaminated soil environments and is well known for its tolerance and degradation capacity for organic solvents (1). Here, we report a draft genome sequence of an organophosphate-degrading strain of *P. putida*, isolated from ranch soil in Fairchild, TX, through an Environmental Sampling Research Module undertaken by University of Houston biotechnology undergraduates (Houston, TX) (2). The closest relative to CBF10-2 is the naphthalene degrader *P. putida* CVS86, known for its preference for the polycyclic aromatic naphthalene as a carbon source over glucose (3). While appearing to lack a clearly defined naphthalene operon itself, CBF10-2 shares its species’ proclivity for aromatic hydrocarbon substrates and possesses an assortment of degradation enzymes with broad activity against petroleum hydrocarbons as well as organophosphate phosphotriesters and phosphorothioates. The genome sequencing of CBF10-2 was performed through Illumina MiSeq paired-end sequencing (35 to 251 bp in each read), with a final sequencing coverage of 61×. Sequence reads were assessed for quality using FastQC (4) and filtered using BBTools (5), with a minimum Phred score of 20. Paired-end reads were assembled into 73 contigs with the SPAdes 3.7 program (6). Preliminary reference-based annotation using PATRIC (7) Web resources was carried out to identify conserved pathways. Final *de novo* annotation was performed with Prokka (8) and the NCBI Prokaryotic Genome Annotation Pipeline (http://www.ncbi.nlm.nih.gov/genomes/static/Pipeline.html). The metabolic pathways of aromatic and heterocyclic compounds were examined using the KEGG databases (9). This draft genome of strain CBF10-2 consists of a total of 6,120,625 bp. CBF10-2 contains 5,449 total genes, of which 64 are pseudogenes, 1,140 represent hypothetical proteins, and 4,171 form known functional proteins. The genome has a G+C content of 63.72% and contains nine rRNA (five complete and four partial), 61 tRNA, and four noncoding RNA (ncRNA) loci.

### Nucleotide sequence accession numbers.

The *Pseudomonas putida* CBF10-2 whole-genome shotgun (WGS) project has the project accession no. LUCV00000000. This version of the project (01) has the accession number LUCV01000000 and consists of sequences LUCV01000001 to LUCV0100073.
